# Reactive in-stent stenosis of a pipeline embolization device in a child: A case report

**DOI:** 10.1097/MD.0000000000018092

**Published:** 2019-11-22

**Authors:** Ting Wang, Chang Wei Zhang, Seidu A. Richard, Wang Chaohua, Xiao Dong Xie

**Affiliations:** a Department of Neurosurgery, West China Hospital, Sichuan University, Chengdu, 610041, P. R. China; b Department of Medicine, Princefield University, Ho-Volta Region, Ghana, West Africa.

**Keywords:** ACA, child, dizziness, ISS, Neuroform, PLED

## Abstract

**Rationale:**

: Pipeline embolization device pipeline embolization device (PLED) is one of the most preferred flow-diverting devices used in treating giant and complex intercranial aneurysms. The occurrence of in-stent stenosis (ISS), which is a partially reversible complication, has been associated with PLED. Trauma around the neck of the aneurysm during our attempts to implant the PLED across the aneurysm resulted into inflammatory reactions, endotheliazation, granular tissue formation, and subsequent ISS.

**Patient concerns:**

We present an 11-year-old girl with dizziness of 6 days duration on account of which she was admitted at our institution. Physical as well as neurological examinations did not yield much.

**Diagnoses:**

Cerebral angiography revealed a right cavernous segment giant aneurysm.

**Interventions:**

We initially implanted Pipeline embolization devices (PLEDs) (ev3, Irvine, California, USA) across the neck of the aneurysm which resulted into ISS 6 months after the operation.

**Outcomes:**

We also attempted balloon angioplasty which failed during our second operation. She was finally treated with Neuroform stent (Stryker Neurovascular, USA) with no further complication and two years follow-up revealed no ISS.

**Lessons subsections as per style:**

A combination of multiple kinds of flow diverting devices could reduce the incidence of ISS in selected patients with complex aneurysms. Minimal trauma caused by PLED at aneurysm site could also reduce incidence of ISS.

## 1 Introduction

Pipeline embolization device (PLED) is one of the most preferred flow diverting devices used in treating giant and complex intercranial aneurysms.^[1–3]^ Ischemia as a result of parent vessel occlusion, thromboembolism, intraparenchymal hemorrhage as well as aneurysm rupture are the potential complications associated with PLED.^[2]^ Nevertheless, the incidence of in-stent stenosis (ISS), which is a partially reversible complication has been associated with PLED.^[1,3]^ In most cases, ISS is usually observed as a tardy complication.^[3]^ Segmental, diffuse as well as multifocal ISS is usually associated with PLED.^[3]^ The pathophysiology of ISS although still a matter of debate is a complex biological process.^[1,4]^ Angiography is usually the most preferred modality used to follow patient who are treated via PLED.^[3,5,6]^ ISS is often asymptomatic and if at all symptomatic, signs and symptoms are observed two months after the initial operation on angiogram.^[2,3]^ Our patient developed ISS after our operation with PLED. She was finally treated with Neuroform stent (Stryker Neurovascular, USA) with no further complications.

## 2 Case report

We present an 11-year old girl with dizziness of six days duration on account of which she was admitted at our institution. Physical as well as neurological examinations did not yield much. Cerebral angiography revealed a right cavernous segment giant aneurysm (Fig. 1  A). We managed the patient via endovascular therapeutic option. The entire endovascular procedure was done under general anesthesia. Implantation of PLEDs (ev3, Irvine, California, USA) across the aneurysm necks was the best endovascular treatment option for this patient. Our aim was to make sure the PLED bridged the aneurysm necks. We introduced a 7 French 90 cm cook long sheath (eV3) and a 5 French 115 cm Navien intermediate catheter (eV3) into the aneurysm to provide support for the pipeline delivery and releasing. The first pipeline was released successfully and the microcatheter went through the first pipeline smoothly (Fig. 1  B).

**Figure 1:**
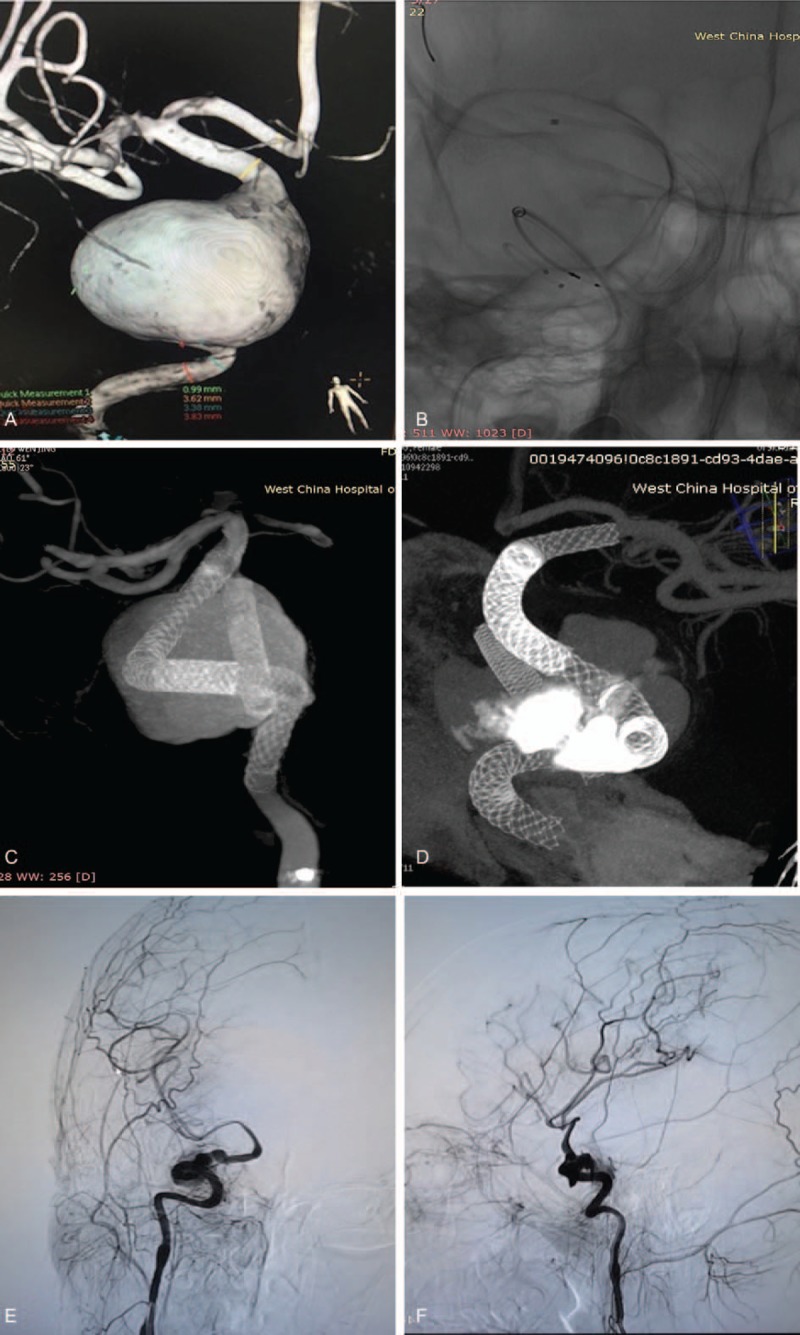
A, Cerebral angiography showing a right cavernous segment giant aneurysm. B, This figure shows a successful implantation of the first pipeline. The microcatheter also went through the first pipeline smoothly. C, This figure shows a reconstructed parent vessel after implantation of pipelines. D, This figure shows no stenotic changes and a normal reconstructed segment of the vessel 3 months after the procedure, E, and F, The vertebral artery and contralateral ACA compensate the right middle communicating artery (MCA) and right ACA through the posterior communicating artery (PCA). G, H, and I, These figures show ISS at bridging points of the PLED six months after the procedure. J, This figure shows successful implantation of Neuroform stents in the stenotic point (ISS). K, This figure shows a visible ipsilateral ACA with improved blood flow and speed. M, No ISS two years after implantation of the Neuroform stent.

**Figure 1 (Continued):**
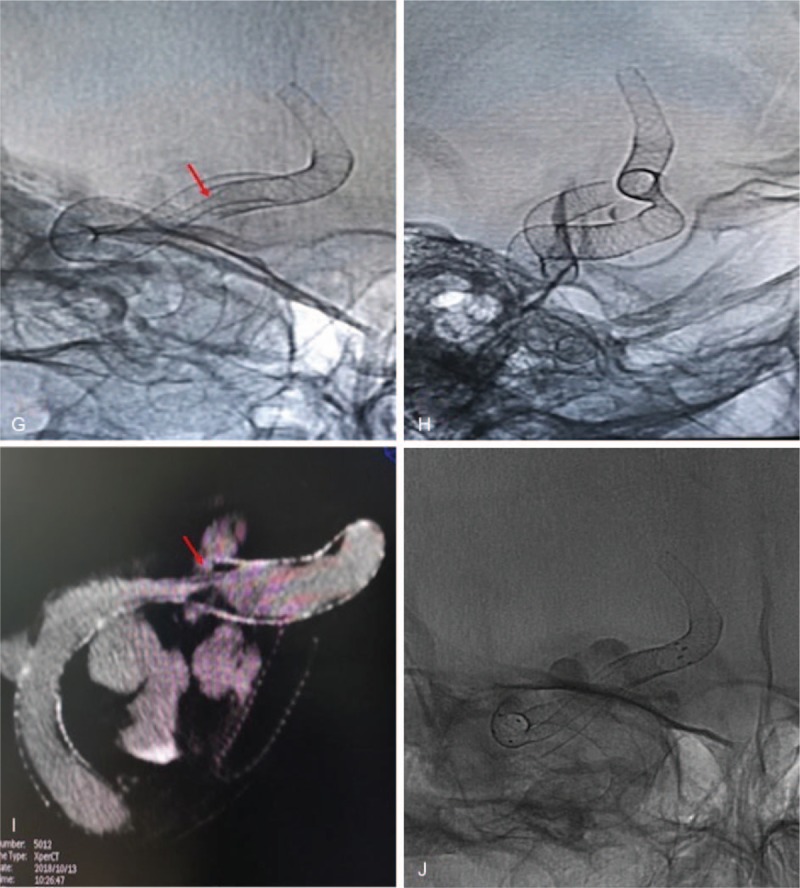
A, Cerebral angiography showing a right cavernous segment giant aneurysm. B, This figure shows a successful implantation of the first pipeline. The microcatheter also went through the first pipeline smoothly. C, This figure shows a reconstructed parent vessel after implantation of pipelines. D, This figure shows no stenotic changes and a normal reconstructed segment of the vessel 3 months after the procedure, E, and F, The vertebral artery and contralateral ACA compensate the right middle communicating artery (MCA) and right ACA through the posterior communicating artery (PCA). G, H, and I, These figures show ISS at bridging points of the PLED six months after the procedure. J, This figure shows successful implantation of Neuroform stents in the stenotic point (ISS). K, This figure shows a visible ipsilateral ACA with improved blood flow and speed. M, No ISS two years after implantation of the Neuroform stent.

**Figure 1 (Continued):**
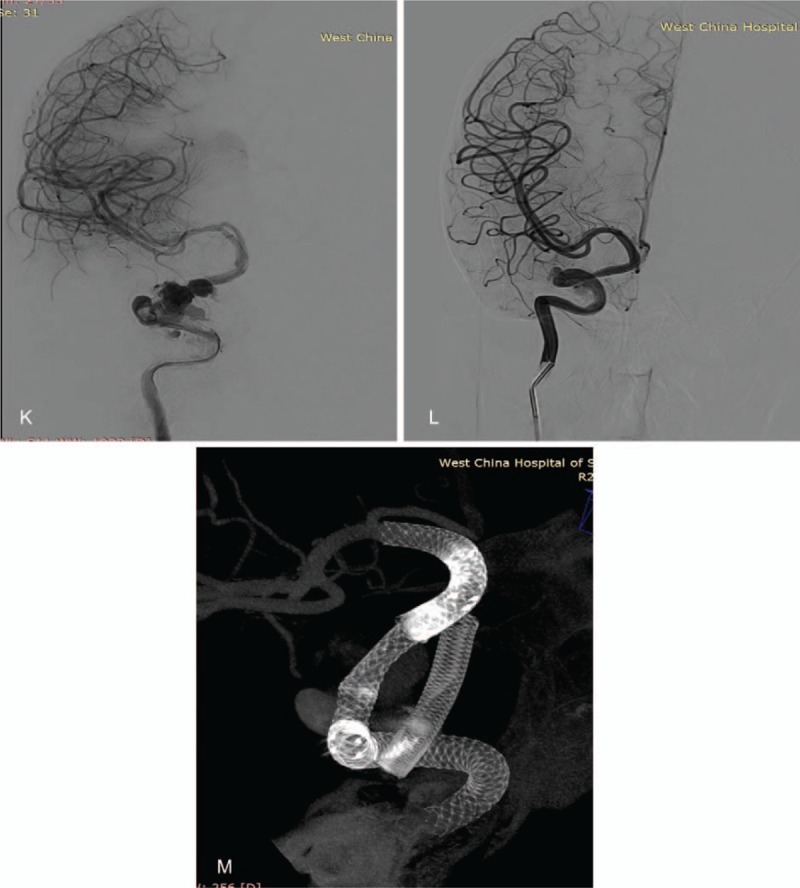
A, Cerebral angiography showing a right cavernous segment giant aneurysm. B, This figure shows a successful implantation of the first pipeline. The microcatheter also went through the first pipeline smoothly. C, This figure shows a reconstructed parent vessel after implantation of pipelines. D, This figure shows no stenotic changes and a normal reconstructed segment of the vessel 3 months after the procedure, E, and F, The vertebral artery and contralateral ACA compensate the right middle communicating artery (MCA) and right ACA through the posterior communicating artery (PCA). G, H, and I, These figures show ISS at bridging points of the PLED six months after the procedure. J, This figure shows successful implantation of Neuroform stents in the stenotic point (ISS). K, This figure shows a visible ipsilateral ACA with improved blood flow and speed. M, No ISS two years after implantation of the Neuroform stent.

We noticed displacement of first pipeline into the aneurysm sac during our attempt to introduce the second pipeline. So, we withdrew the second pipeline and attempted several times to reposition the first pipeline cross the aneurysm necks with hitches. We finally reconstructed the aneurysmal artery with three PLEDs (Fig. 1  C). The patient was put on dual-antiplatelet therapy comprising of daily soluble aspirin and clopidogrel according to her body mass index after the operation. She was discharged home 5 days after the operation.

Three months follow-up revealed a decrease in the aneurysm sac with satisfactory ipsilateral anterior communicating artery (ACA) opacification but a decreased blood flow. On angiogram, the conformation of the stent in Towne's and lateral views revealed no stenotic changes and reconstructed segment still normal (Fig. 1  D) Also, the conformation in bridging point looks normal and smooth without ISS.

The six-month follow-up revealed a smaller aneurysm sac and a further decrease in blood flow. The ipsilateral ACA opacification was no longer visible. We observed that, the vertebral artery and contralateral ACA compensated the right middle communicating artery (MCA) and right ACA through the posterior communicating artery (PCA) (Fig. 1  E and 1F). We also observed ISS at bridging points of the PLED (Fig. 1G, 1H and 1I). We attempted retreating the patient via balloon angioplasty but failed to achieve a satisfactory blood flow and speed.

We finally treated the patient with Neuroform stent (Stryker Neurovascular, USA). The stent was implanted in the stenotic point (Fig. 1  J). The blood flow and speed improved and the ipsilateral ACA was visible again (Fig. 1  K). Again, after the second procedure, the patient put on dual-antiplatelet therapy comprising daily soluble aspirin and clopidogrel according to her body mass index after the operation. Two years follow-up revealed no ISS (Fig. 1  M).

## 3 Discussion

Pipeline embolization device (PLED) have proven to be effective in the management of wide necked or complex fusiform aneurysms.^[1,5]^ PLEDs are able to occlude the aneurysm by initiating intra-aneurysmal thrombosis. The occlusion of the aneurysm sac occurs as a result of PLED's ability to disrupt blood flow into the aneurysm sac.^[3,7]^ Nevertheless, PLED are not devoid of complications such as ISS. Our patient is the second pediatric case who developed ISS during management with PLED. Almost all the cases reported in literature are adult cases. However, the incidence of age variance in relation to this endovascular complication has not been exploited.

Most of the complications as observed by Cohen et al were grouped into stent configuration and location.^[3]^ These changes were delayed end tapering, configuration changes as well as minor and major creeping.^[3]^ Signs of minor creeping were observed on the stent after implant as variations in marker disposition while major creeping were observed as variations in the absolute location of the stent ends compared to original placement or even migration.^[3]^

Also, several animal prototypes have been used to demonstrate diverse phases of cellular reaction after stent placements resulted into ISS.^[1]^ Schwartz et al classified these cellular reactions into early, intermediate and late phases.^[6]^ The early phase consists of thrombus development as well as inflammation while the intermediate phase consists of endothelialization as well as granulation tissue formation.^[1,6]^ The late phase however consists of smooth muscle cells as well as matrix development.^[1,6]^ We think trauma around the neck of the aneurysm during our attempts to implant the PLED across the aneurysm resulted into inflammatory reactions, endotheliazation, granular tissue formation, and subsequent ISS.

Xiang et al are of the view that torpid aneurysmal flow as well as extremely low wall shear pressure may stimulate aneurysmal wall mortification through unknown inflammatory pathways leading to ISS.^[4]^ Cohen et al are of the view that immediate angiographic manifestation of ISS and improvement of patients whose antiplatelet agents’ doses were augmented suggest a distinctive biological behavior that is very dissimilar to the ISS that was initially related to neointimal hyperplasia.^[3]^

Lubicz et al evaluated ISS by assessing the gap between the contrast filled vessel lumen and the inner contour of the stent,^[7]^ while Ruben et al evaluated ISS by linking vessel lumen on follow-up with the initial angiograms.^[1]^ We adapted both evaluation modalities as used by Lubicz et al and Ruben et al assess ISS in our case. In most ISS cases, stenosis is usually asymptomatic. During follow-up after our initial treatment, our patient did not show any symptoms.

In severe ISS cases, antiplatelet therapy has proven to be effective in reversing the stenosis with only conservative management.^[3]^ In our case however, although we gave aspirin and clopidogrel, a second stenting with Neuroform stent was necessary to ensure blood flow and speed was adequate in the ipsilateral ACA. Balloon angioplasty was not a good treatment option for our patient. Cohen et al established that only angioplasty balloons and not low-pressure balloons are capable of reopening tapered diverter ends that are implanted in the arterial wall.^[3]^ We however did not attempt diverter angioplasty during the management of our patient.

Several studies have proven that Neuroform stent accelerates the endovascular management of multifaceted and wide-necked cerebral aneurysms.^[8–10]^ Also, Neuroform stents may strengthen the robustness of aneurysm embolization as a result of amalgamation of hemodynamic as well as biological consequences.^[8,11]^ Canton et al proved that Neuroform stents, notwithstanding their trifling metal surface area coverage, can considerably change the baseline hemodynamics.^[11,12]^ These stents were able to decrease the velocity of aneurysmal inflow jet as well as decrease intra-aneurysmal vorticity and shear pressure.^[8,11,12]^ Nevertheless, like any flow diverting device, Neuroform also has complications like thromboembolism and delayed ISS.^[8]^ We achieved adequate blood flow and speed in the ipsilateral ACA using Neuroform stents with no further complications.

## 4 Conclusion

The choice of flow diverting devices should be individualist with much attention paid to the location, size as well as the nature of the aneurysm. A combination of multiple kinds of flow diverting devices could reduce the incidence of ISS in selected patients with complex aneurysms. Minimal trauma caused by PLED at aneurysm site could also reduce incidence of ISS.

## Author contributions

**Conceptualization:** Ting Wang, Chang Wei Zhang, Seidu A Richard, Wang Chaohua, Xiao Dong Xie.

**Data curation:** Ting Wang, Chang Wei Zhang, Seidu A Richard, Wang Chaohua, Xiao Dong Xie.

**Formal analysis:** Ting Wang, Wang Chaohua, Xiao Dong Xie.

**Funding acquisition:** Chang Wei Zhang.

**Methodology:** Chang Wei Zhang, Seidu A Richard, Xiao Dong Xie.

**Resources:** Ting Wang, Wang Chaohua.

**Supervision:** Chang Wei Zhang.

**Writing - Original Draft:** Ting Wang, Seidu A Richard.

**Writing - Review & Editing:** Ting Wang, Chang Wei Zhang, Seidu A Richard, Wang Chaohua, Xiao Dong Xie.

## References

[1] Mühl-Benninghaus R Haußmann A Simgen A Transient in-stent stenosis: a common finding after flow diverter implantation. Journal of Neurointerventional Surgery 2019;11:196–9. 29970620 10.1136/neurintsurg-2018-013975

[2] Miller TR Jindal G Gandhi D Focal, transient mechanical narrowing of a pipeline embolization device following treatment of an internal carotid artery aneurysm. Journal of Neurointerventional Surgery 2015;7:e35–40. 25280566 10.1136/neurintsurg-2014-011384.rep

[3] Cohen JE Gomori JM Moscovici S Delayed complications after flow-diverter stenting: reactive in-stent stenosis and creeping stents. Journal of Clinical Neuroscience 2014;21:1116–22. 24524952 10.1016/j.jocn.2013.11.010

[4] Xiang J Ma D Snyder KV Increasing flow diversion for cerebral aneurysm treatment using a single flow diverter. Neurosurgery 2014;75:286–94. 24867201 10.1227/NEU.0000000000000409

[5] Becske T Potts MB Shapiro M Pipeline for uncoilable or failed aneurysms: 3-year follow-up results. Journal of Neurosurgery 2017;127:81–8. 27739944 10.3171/2015.6.JNS15311

[6] Schwartz RS Chronos NA Virmani R Preclinical restenosis models and drug-eluting stents: still important, still much to learn. Journal of the American College of Cardiology 2004;44:1373–85. 15464316 10.1016/j.jacc.2004.04.060

[7] Lubicz B Collignon L Raphaeli G Flow-diverter stent for the endovascular treatment of intracranial aneurysms: a prospective study in 29 patients with 34 aneurysms. Stroke—A Journal of Cerebral Circulation 2010;41:2247–53. 10.1161/STROKEAHA.110.589911 20798369

[8] Fiorella D Albuquerque F Woo H Neuroform stent assisted aneurysm treatment: evolving treatment strategies, complications and results of long term follow-up. Journal of Neurointerventional Surgery 2010;2:16–22. 21990553 10.1136/jnis.2009.000521

[9] Lylyk P Ferrario A Pabón B Buenos Aires experience with the Neuroform self-expanding stent for the treatment of intracranial aneurysms. Journal of Neurosurgery 2005;102:235–41. 15739550 10.3171/jns.2005.102.2.0235

[10] Jankowitz BT Hanel R Jadhav AP Loy DN Frei D Siddiqui AH Neuroform Atlas Stent System for the treatment of intracranial aneurysm: primary results of the Atlas Humanitarian Device Exemption cohort. Journal of neurointerventional surgery. 2019:neurintsurg-2018-014455. 10.1136/neurintsurg-2018-014455 PMC6703120 30670625

[11] Cantón G Levy DI Lasheras JC Hemodynamic changes due to stent placement in bifurcating intracranial aneurysms. Journal of Neurosurgery 2005;103:146–55. 16121985 10.3171/jns.2005.103.1.0146

[12] Cantón G Levy DI Lasheras JC Flow changes caused by the sequential placement of stents across the neck of sidewall cerebral aneurysms. Journal of Neurosurgery 2005;103:891–902. 16304994 10.3171/jns.2005.103.5.0891

